# Closed reduction, internal fixation with quadratus femoris muscle pedicle bone grafting in displaced femoral neck fracture

**DOI:** 10.4103/0019-5413.38578

**Published:** 2008

**Authors:** Sibaji Chaudhuri

**Affiliations:** Consultant Orthopaedic Surgeon, Former Registrar of Orthpaedic Surgery, Bardhaman Medical College and R.G.Kar Medical College, Kolkata - 700 004, India

**Keywords:** Avascular necrosis of femoral head, closed reduction internal fixation, femoral neck fracture, muscle pedicle bone graft, quadratus femoris muscle pedicle bone grafting

## Abstract

**Background:**

Management of femoral neck fracture is still considered as an unsolved problem. It is more evident in displaced fractures where this fracture is considered as some sort of vascular insult to the head of the femur. We have used closed reduction, internal fixation and quadratus femoris muscle pedicle bone grafting in fresh displaced femoral neck fractures.

**Materials and Methods:**

From April 1996 to December 2004 we operated 73 consecutive patients of displaced femoral neck fracture in the age group of 24 to 81 years, mean age being 54.6 years. The patients were operated within one week of injury, the mean delay being 3.6 days. Closed reduction internal fixation along with quadratus femoris muscle pedicle bone grafting was done in all cases. They were followed up for an average period of 5.6 years (range 2-11 years).

**Results:**

Results were assessed according to modified Harris Hip Scoring system and found to be excellent in 53, good in 12, fair in six and poor in two patients. Bony union occurred in 68 cases, no patient developed avascular necrosis (AVN) till date.

**Conclusion:**

For fresh displaced femoral neck fracture in physiologically active patients closed reduction, internal fixation and quadratus femoris muscle pedicle bone grafting is a suitable option to secure union and prevent development of AVN.

## INTRODUCTION

Ever since Von Langenback^1^ reported in 1878 open reduction and internal fixation for fracture neck of femur by using silver pins, various authors postulated different methods but the ideal goal is still not achieved. Till today there is no satisfactory solution in the management of femoral neck fracture so far as union of the fracture and avascular necrosis of femoral head is concerned. The best hip is obtained after femoral neck fracture when the fracture heals in normal anatomical alignment and at the same time escapes avascular necrosis (AVN) and late segmental collapse.

Displaced femoral neck fracture is considered as a vascular injury to the head of the femur. Marry Catto^2^ in a histological study of 109 femoral heads, removed more than 16 days after fracture showed all of these suffered some damage to vascular supply. Sudhir Kumar *et al.*,^3^ with the use of Tetracycline labeling showed that the chance of femoral head being vascular was 50% in more than three weeks' old untreated fractures of femoral neck.

For the same reason the surgeon has less control over AVN than in the case of union. The incidence of nonunion varies in the literature from 0-22% whereas incidence of AVN is 5.5-70% in various series.^4^–^9^

The vascularised graft like quadratus femoris muscle pedicle bone graft as used in Meyers^8^ procedure might solve the problem of AVN. In Meyers procedure of open reduction and internal fixation with quadratus femoris muscle pedicle bone grafting, there is a risk of compromising the medial circumflex femoral artery which runs along the post aspect of the base of the neck. To avoid this we have reduced the fracture by closed method and after fixation of fracture by two or three Knowle's pins/ or cannulated hip screws, opened the capsule in the middle of the subcapital region and introduced the quadratus femoris muscle pedicle bone graft in a slot prepared in the head of the femur. We present an analysis of 73 cases treated by closed reduction internal fixation and quadratus femoris-based muscle pedicle bone graft.

## MATERIALS AND METHODS

We treated 73 consecutive cases of fresh displaced femoral neck fractures (Garden Stage III and Stage IV) by closed reduction internal fixation and muscle pedicle bone grafting between April 1996 and December 2004. Age of the patients varied from 24 years to 81 years (mean age 54.6 years). Forty-six patients were male and 27 patients were female. The inclusion criteria were: patients who were physiologically active, had good osteoporotic index (Singh's index^10^ 6, 5 or 4), were anesthetically fit to be taken in prone position and fresh cases presenting within seven days of trauma were included in study.

Thirty-two patients sustained road traffic accident of which 13 cases were due to high-energy trauma, 41 patients sustained fall at their residence. Pathological fractures, patients with life expectancy less than five years and associated systemic pathology like ankylosing spondylitis were excluded from the study. One patient had associated ipsilateral compound fracture both bones leg and fracture shaft of the femur and femoral neck fracture.

### Surgical procedure

Following regional or general anesthesia the patient was placed on the fracture table in prone position. Reduction was done in extension. The reduction was checked under image intensifier in both AP and lateral views. The Garden alignment index for assessing quality of reduction was followed rigidly.^11^

**Figure 1 (a-d):**
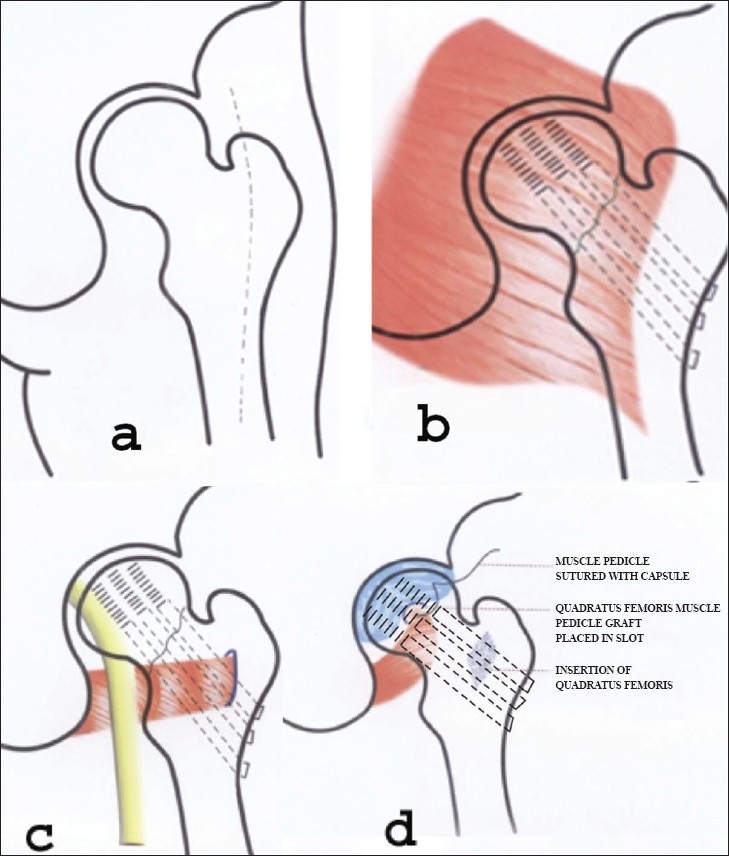
Line diagram showing incision (a), internal fixation before splitting the gluteus maximus (b), position of quadratus femoris (c) and anchoring of quadratus femoris muscle pedicle bone graft (d)

An 11 to 13 cm long incision was made starting from just above and medial to the tip of the greater trochanter and then along the greater trochanter to the shaft of the femur [Figure 1a]. After separating the deep fascia and splitting the vastus lateralis, the base of the trochanter and upper shaft of femur was exposed. Three Knowles pins/cannulated hip screws were inserted parallel to each other in AP view starting from just above the level of the calcar and going above it subsequently [Figure 1b]. In the lateral view pins were placed in more or less central position of head and neck so as to keep enough room for the placement of muscle pedicle bone graft in the posterior aspect of head. After fixation of the fracture the vastus lateralis was closed, and gluteus maximus was splitted.

The rectangular graft was then marked out at the insertion of the quadratus femoris in the trochanteric crest of the femur with a small osteotome [Figure 1c]. The graft started 1 to 2 cm distal to the tip of the greater trochanter and came down along the insertion of the quadratus femoris and ended just above the level of the lesser trochanter. Drill holes were made along the outline of the graft. Then with straight and curved osteotome a graft of 1 cm depth and 1 cm width was gently cut out, a graft of this size always keeps the posterior vascular anastomotic circle safe. Its length varied according to the width of the quadratus femoris. In our series it was about 3 cm in length. The graft was slightly mobilized by finger dissection and retracted downward and medially. Two bone levers were now placed on either side of the neck of femur extracapsularly to visualize the capsule. The medial circumflex femoral artery could be seen and felt at the base. Position of placement of the graft was checked under image intensifier.

The posterior capsule was visualized and incised in the middle of the subcapital region, this also safeguarded the lateral epiphiseal vessel and practically caused no damage to the ascending cervical arteries, the position of which was further confirmed under image intensifier by placing a haemostat over the head of the femur. A slot was made for the graft with a small osteotome and gouge. The cephalad end of the graft was made slightly tapered and was introduced in the slot. The post capsule was then stitched with quadratus femoris and the graft was found firmly secured in the slot [Figure 1d]. The width of the quadratus femoris graft should not be too wide otherwise it may endanger the artery. In 22 cases additional screw fixation was required for securing the graft. Gel foam was applied over donor site at trochanteric crest. The wound was closed in layers over a suction drain.

This procedure did not require blood transfusion unless the patients were already anemic and in our series only six patients needed blood transfusion.

The patients were allowed to sit up in bed after 24 h and were allowed non weight bearing crutch walking after two weeks. The patients were followed up at six weeks, nine weeks and 12 weeks, then every eight to 12 weeks for one year and then every three to six months till two years, after that every year.

At follow-up they were asked to do active straight leg raising only after 12 weeks but active and assisted active exercises of hip were encouraged.

## RESULTS

The follow-up period varied from two years to 11 years (average being 5.6 years). From 12 weeks onwards the patient was asked to come to the clinic for evaluation and recording of clinical examination, range of movements, pain on weight bearing, limp and leg length discrepancy if any. Anteroposterior and lateral view X-rays were also taken at that time. Satisfactory bony union, defined as the presence of bony continuity across the fracture gap and absence of pain on weight bearing were seen in 68 out of 73 patients and the fracture united in 14 to 18 weeks' time giving a union rate of about 93%. Thirteen patients were in the age group of 24 to 40 years, all of them were male patients and victims of high-energy trauma. In all of them the fracture united and none of them showed any evidence of AVN in an average 5.6 years of follow-up. In this young age group some restriction of hip rotation movements persisted in most of the cases.

**Figure 2 (a-d):**
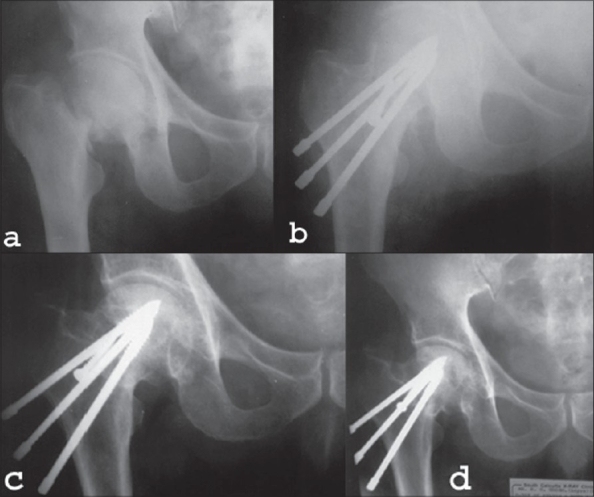
X-ray anteroposterior view (a) showing femoral neck fracture in an 81-year-old male patient (b) and immediate postoperative X-ray of the same patient. The muscle pedicle was fixed by a screw. (c) X-ray anteroposterior view of same patient showing six months follow-up (d) and five years follow-up. The pt showed excellent clinical outcome

In 43 patients there was some shortening, five patients in whom the fracture remained ununited there was 1.5 to 2 cm shortening. In the remaining 38 patients there was only. 5 cm to 1 cm shortening. In one patient of road traffic accident with fracture of both bones of leg and fracture shaft of femur, the fracture neck of the femur of the ipsilateral side was diagnosed only at check X- ray. The patient was operated upon two days later by the same procedure and went into union. Shortening was due to collapse at the fracture site [Figure 2 a-d], when we accepted slight valgus reduction within the limits of Garden's alignment index we did not get any shortening, in the younger age group anatomical reduction also did not result in any shortening (Table 1).

**Table 1:** Clinical details of the patients and the results

Age (Years)	Gender		Mean Period	Mean time	NU	AVN	Coxa vara	Pin/screw	Shortening		
			of delay (days)	of union (weeks)				backing out			
	Male	Female							Nil	5-1 cm	>1 cm
20 to 40	13	0	3.5	14	0	0	0	2	11	2	0
41 to 50	4	2	3.1	14.6	0	0	0	3	3	3	
51 to 60	9	19	4.2	15.4	2	0	2	18	10	16	2
61 to 70	15	6	3.1	16.2	1	0	1	15	6	14	1
71 and above	5	0	3.6	17.2	2	0	2	5	0	3	2

AVN - Avascular necrosis, Nu - Non union, Cm - Centimeter

Superficial infection occurred in four cases which healed within 15 days by regular dressing and broad-spectrum antibiotic therapy. In one patient who developed co-morbidity in the form of hematemesis and renal failure deep infection persisted, implants were taken out. The result was poor. From one year onwards we looked for any evidence of aseptic necrosis both clinically and radiologically. In two to 11 years' follow-up we did not get a single case of AVN.

**Figure 3 (a-d):**
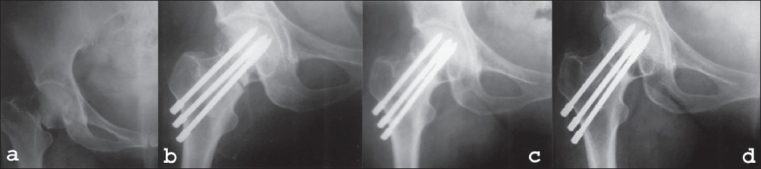
X-ray anteroposterior view (a) showing femoral neck fracture in a 49-year-old female patient (b) and immediate postoperative X-ray of the same patient, (c) X-ray anteroposterior view of the same patient at follow-up at four months (d) and at two years shows fracture consolidation and absence of AVN

At the end of two years results were analyzed according to modified Harris hip scoring^12^,^13^ system (Table 2) and it was found to be excellent in 53 [Figure 3], good in 12, fair in six and poor in two patients. Four patients of the nonunion group did not want any further intervention and could manage their activities of daily living very well and needed a cane only during prolonged walking. One patient who had developed co-morbidity really needed total hip arthroplasty but she and her family refused further surgery considering her co-morbid conditions.

**Table 2:** Grading according to modified Harris hip score^12^,^13^

Age (Years)	Grading according to modified Harris hip score*			
	
	E	G	F	P
20 to 40	13			
41 to 50	5	1		
51 to 60	18	6	4	
61 to 70	16	3	1	1
71 and above	1	2	1	1

* E- excellent 53, G - good 12, F - fair six and P - poor two

## DISCUSSION

Femoral neck fracture disrupts femoral head perfusion in multiple ways. Displaced cervical fractures disrupt all intraosseous supply from the neck leaving only surviving subsynovial ascending arteries and contribution from ligamentum teres (if present) to nourish the head. There is a consensus opinion that in physiologically active patients we should try to preserve the head of the femur. Early operation and stable internal fixation by multiple pins/screws has increased the rate of union remarkably well during the last century. Deyerle,^14^ Swiontkowski^6^ have shown 90 to 100% union rate in their cases but incidences of AVN continued to be high and more so in younger patients. Protzman and Burkhalter^15^ in a review of 22 young patients in 1976 showed 86% incidence of AVN.

Search continued to identify the fractures heading towards AVN of femoral head. Hirata, Tetsuo *et al.*,^16^ used dynamic magnetic resonance imaging in 36 cases of femoral neck fractures within 48 h of injury and found absence of femoral head perfusion in 19 patients. In these group of 19 patients, osteonecrosis developed in 10 patients and nonunion developed in five patients. Cho, Myung-Rae *et al.*,^17^ conducted a study from March 1999 to January 2001 on 44 patients (mean age 51 years, range 18-76 years) on all four Garden's Stages of fractures. The average delay between injury and surgery was 52 h. Presence of bleeding from drill holes used for cannulated screw placement was observed for 5 min. In six cases there were no bleeding and all of them developed AVN in subsequent follow-up, one patient in the bleeding group also developed AVN. From the last two observations and also from Swiontkowski's^6^ observation it can be ascertained that urgent operation doesn't give guarantee against AVN.

Judet^18^ tried quadratus femoris muscle pedicle bone graft in dogs and later used the same technique over human being. Day, Brian *et al.*,^19^ used iliopsoas muscle pedicle bone graft in dogs. The microangiographic and histological studies showed the procedure could maintain the vascularity and viability of the femoral head.

Meyers^8^ technique of open reduction and internal fixation may endanger the medial circumflex femoral artery. In transcervical fractures, the fracture line itself is very near the artery. In Meyers series^8^ the rate of union was 89% and the incidence of AVN was 8% only which was remarkable at that time. In our series to avoid any injury to the medial circumflex femoral artery we did closed reduction and internal fixation and then did muscle pedicle graft by opening the capsule in the middle of the head neck junction. Grant's^20^ atlas of anatomy was strictly adhered to during our operative procedure. After harvesting the quadratus femoris and mobilizing it we just retracted gemelli and obturator internnus to reach the capsule. Gautier's^21^ description of the medial circumflex femoral artery also explains that exposing the capsule in its middle at the head neck junction safeguards both the main vessel and its important superior retinacular arteries. Mere opening of the capsule does not do any harm to the fracture or convert this procedure into open reduction. Swiontkowski^6^ in his series of urgent operation in young patients always opened the capsule to reduce any tamponade affect and sometimes palpated the fracture line to be very sure about reduction. In our series we never tried to palpate the fracture line even in subcapital fractures.

Fixation of the fracture should preferably be done with three pins. In our initial cases to accommodate the quadratus femoris muscle pedicle graft fixation screw, we had used two pins only in 15 cases. Maurer *et al.*,^22^ in a cadeveric study showed that two screws may be an acceptable fixation. In Meyers^8^ article it was said that the quadratus femoris may be absent in about one per cent cases. During this study we never came across this. The older age group (71 plus) patients were included in this study after properly counseling them.

Some of the results of previous authors doing closed reduction and internal fixation were highlighted in the introduction. In a more recent study by Nikolopoulos *et al.*,^23^ with a patients' profile very similar to the present study (mean age 58 years, mean injury and operation delay 5.3 days) treatment was done by closed reduction and internal fixation. Incidence of AVN occurred in 15 cases out of 38 patients of Garden Stage III and IV (40%). Sud *et al.*,^24^ by using closed reduction percutaneous cannulated screw fixation reported 22% nonunion in displaced fractures and 10% cases of AVN in a series comprising 60% displaced fractures. In our series union rate was significantly higher in the less than 71 years age group patients and there were no incidences of AVN in all age groups. In our view this was due to closed reduction internal fixation and opening of the capsule at the middle of the head-neck junction after properly visualizing the medial circumflex femoral artery on the posterior aspect of the base of the neck. But whether this procedure can prevent AVN or not can only be ascertained by reproducing the results in the hands of other surgeons, using the same procedure for a considerable period of time. Long-term results of bipolar hemiarthroplasty are also not very satisfactory. In a maximum period of four and a half years follow-up only 31% patients showed excellent results in a series of 80 patients, as reported by Asif *et al.*^25^ An orthopedic surgeons' survey on the treatment of displaced femoral neck fractures reported by David Chua *et al.*,^26^ reveals that the majority of surgeons prefer internal fixation for a 70-year-old lady who is otherwise physically fit. On the other hand for a physically unfit old lady of 84 years the majority of surgeons opined for hemiarthroplasty. Juha Partanen *et al.*,^27^ in a matched pair study of 168 cases of displaced femoral neck fracture showed functional ability was better in the osteosynthesis group but reoperation rate was higher compared to the hemiarthroplasty group. Blomfeldt R *et al.*,^28^ in their study on 60 patients with a mean age of 84 years said there was little to recommend to hemiarthroplasty with an uncemented Austin Moore prosthesis compared with internal fixation in patients with cognitive dysfunction, but hip function was better in the internal fixation group.

To conclude closed reduction internal fixation and quadratus femoris muscle pedicle bone grafting is a simple and effective procedure. Taking care of the details of anatomy at the posterior aspect of the neck of the femur this procedure can be easily maneuvered by an average orthopedic surgeon.
